# Modality- and sequence-specific effects of guided imagery on stress and neuroendocrine markers in young adults: A pilot randomized crossover study

**DOI:** 10.1016/j.cpnec.2026.100384

**Published:** 2026-10-06

**Authors:** Nurwina Anuar, Manal M. Bayyat, Ibrahim M. Dabayebeh, Shazli Ezzat Ghazali, Zainab H. Abumuaili, Tala Al-Hadid, Haidar Djemai

**Affiliations:** a School of Sports Sciences, Universiti Kebangsaan Malaysia, Bangi, Selangor, 43600 UKM, Malaysia; b School of Sports Sciences, The University of Jordan, Queen Rania Street (University Street), Jubaiha, Amman, 11942, Jordan; c Faculty of Health Science, Universiti Kebangsaan Malaysia, Bangi, Selangor, 43600 UKM, Malaysia; d School of Medicine, The University of Jordan, Queen Rania Street (University Street), Jubaiha, Amman, 11942, Jordan; e EA 7329, Institute for Research in Bio Medicine and Epidemiology of Sport (IRMES), INSEP, 11, Avenue Du Tremblay, Paris, 75012, France; f INSERM UMR-S 1124, HealthFex, Université Paris Cité, 45 Rue des Saints-Pères, Paris, 75006, France; g Laboratory of Biology and Molecular Genetics, Faculty of Medicine, University Salah Boubenider Constantine 3, New Town Ali Mendjeli, Constantine, Algeria

**Keywords:** Stress reduction, Perceived stress, Cortisol, Serotonin, β-endorphin

## Abstract

**Background:**

Guided mental imagery interventions are increasingly used for stress reduction, but evidence comparing the effects of different imagery modalities remains limited. This pilot study examined modality-specific and sequence-dependent effects of calming and achievement imagery on perceived stress and neuroendocrine markers.

**Methods:**

Thirty-two male adults (18–25 years) completed a 5-day pilot randomized, counterbalanced crossover study. Participants were assigned to calming-then-achievement (CAG, n = 13), achievement-then-calming (ACG, n = 12), or a no-intervention control group (CG, n = 7). Biomarkers were assessed on Days 1, 3, and 5 in active groups and Days 1 and 5 in CG; perceived stress was assessed on Days 1 and 5.

**Results:**

Serotonin was significantly higher following achievement than calming imagery (calming − achievement: −66.5 ng/mL, 95% CI −123.2 to −9.8; *p* = .019; partial η^2^ = .113), with a significant sequence effect (*p* = .014). Cortisol and β-endorphin showed no modality, period, or sequence effects. β-Endorphin increased over time (*p* < .001); cortisol showed no significant time, group, or interaction effects. Serotonin showed significant time, group, and time-by-group effects (all *p* < .001), including a marked increase in the untreated control group by Day 5. No significant effect of sequence or modality was found on perceived stress.

**Conclusions:**

These preliminary, hypothesis-generating results suggest achievement imagery may selectively engage serotonergic activity, while both modalities similarly engage the endogenous opioid system; no effect was found on cortisol or perceived stress. Given the pilot design, small sample, multiple outcomes, and the serotonin increase in the untreated control group, findings should be confirmed in adequately powered randomized trials.

## Highlights

• Achievement imagery increases serotonin more than calming imagery.
• Guided imagery raises β-endorphin regardless of content.
• Short-term guided imagery does not alter cortisol levels.
• Imagery sequence also modulates the serotonin response.
• Perceived stress was unaffected by imagery modality or sequence.

## Graphical abstract

**Figure:**
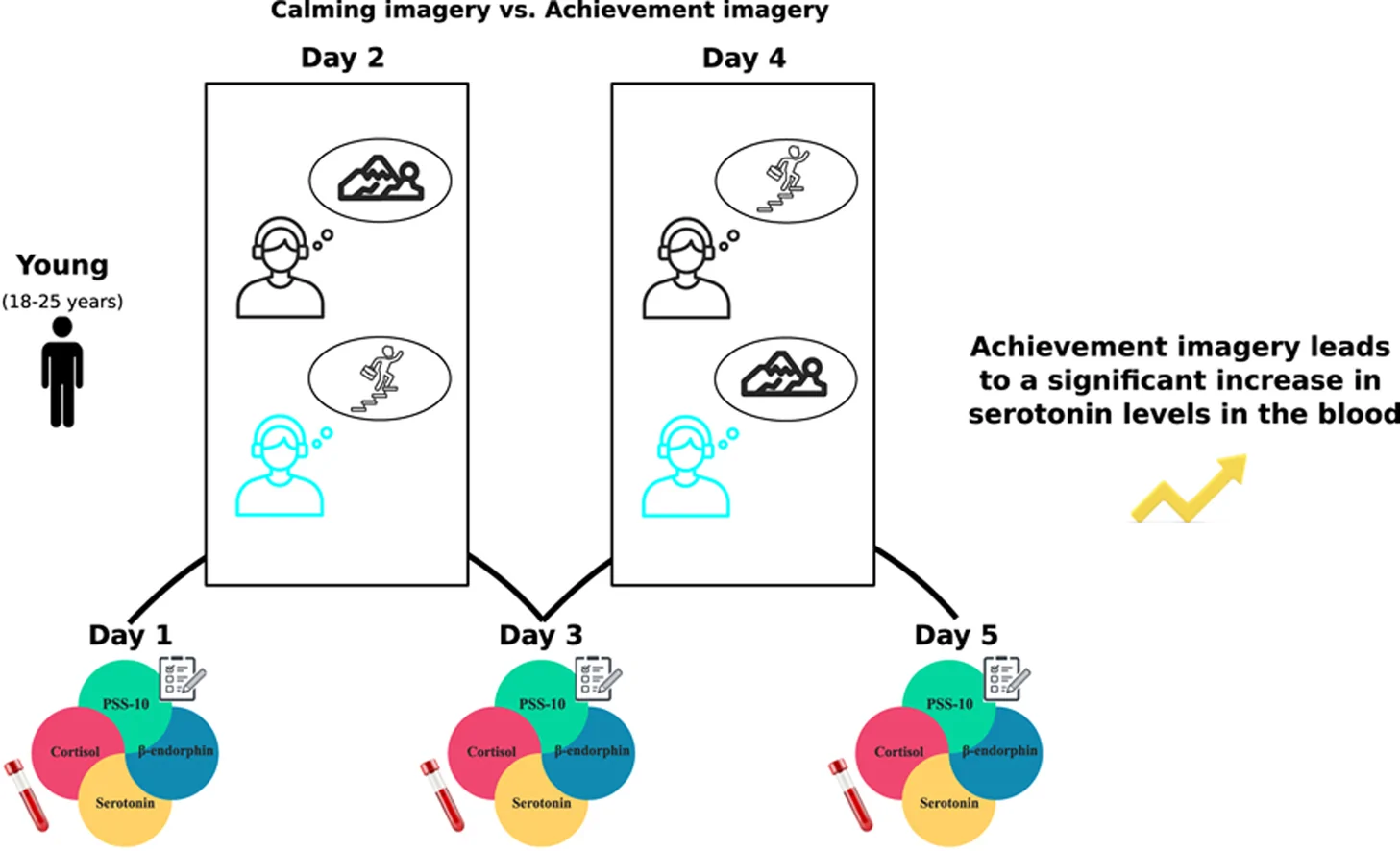

## 1 Introduction

Psychological stress represents one of the most pervasive public health challenges of the 21st century, with profound implications for individual well-being, healthcare systems, and societal productivity [1,2]. Chronic stress exposure is associated with a cascade of adverse health outcomes, including cardiovascular disease, metabolic dysfunction, immune dysregulation, and psychiatric disorders [3]. The hypothalamic-pituitary-adrenal (HPA) axis, the body's primary neuroendocrine stress response system, plays a central role in mediating these effects through the release of cortisol and other stress hormones [4,5]. Beyond the HPA axis, the endogenous opioid system (particularly β-endorphin) and the serotonergic system contribute critically to stress regulation, mood, and emotional resilience [6,7]. Notably, β-endorphins and cortisol (via ACTH) share a common precursor protein called pro-opiomelanocortin (POMC) and are often co-secreted during HPA axis activation in response to stress [8].

Mental imagery, the cognitive process of generating, manipulating, and experiencing sensory representations in the absence of external stimuli, has emerged as a promising non-pharmacological strategy for stress reduction [9,10]. Unlike passive relaxation techniques, mental imagery is an active cognitive process that engages multiple sensory modalities (visual, auditory, kinesthetic, olfactory) and can be tailored to elicit specific psychological and physiological responses [11]. Guided imagery interventions, in which individuals are instructed to vividly imagine specific scenes or experiences, have been shown to reduce subjective stress, anxiety, and depressive symptoms across diverse populations [[12], [13], [14]].

The theoretical foundation for imagery-based interventions is rooted in Lang's [15,16] bio-informational theory, which posits that mental images are structured information networks containing stimulus, meaning, and response propositions. According to Lang [16], imagery that incorporates response propositions information about physiological, affective, and behavioral responses activates the same neural and physiological systems engaged during actual experiences. Brain's sensory pathways handles vivid mental imagery and external stimuli in a similar way; both activate the same topographically organized areas in the brain cortex that are involved in initial perception [10,11]. This framework predicts that different imagery contents should produce distinct psychophysiological effects depending on the specific response propositions embedded in the image.

Recent studies have provided compelling evidence that guided imagery interventions can modulate both psychological and physiological stress markers. Weigensberg's research [17,18] showed a reduction in salivary cortisol from the first 20-min session of guided imagery exposure, however, repeated exposure for 12 weeks altered diurnal salivary cortisol profiles in adolescents, suggesting that regular imagery practice can remodel the basal rhythm of the HPA axis. However, beyond cortisol, guided imagery has been shown to influence autonomic nervous system (ANS) function and other neuroendocrine markers. Toussaint et al. [19] demonstrated that guided mental imagery and progressive muscle relaxation (PMR) produce more consistent immediate physiological effects than deep breathing alone, thus confirming the value of mental imagery as an effective relaxation tool.

Despite accumulating evidence for the efficacy of guided imagery in reducing stress [12,13], a critical gap remains in understanding of how different imagery modalities produce distinct psychophysiological effects. Guided imagery interventions vary widely in content, ranging from calming, nature-based scenes emphasizing peaceful environments such as tranquil beaches or serene forests, to achievement-oriented scenarios focused on goal attainment, athletic performance, academic success, or career accomplishments [20]. These different imagery modalities may engage distinct psychological processes and activate divergent neural circuits, potentially producing different patterns of neuroendocrine response. However, no studies have directly compared the effects of calming versus achievement imagery on objective biomarkers of stress and well-being, leaving clinicians without empirical guidance for selecting optimal imagery content to match specific treatment goals.

Lang's bio-informational theory provides a framework for understanding how imagery content may activate distinct physiological response networks through stimulus, meaning, and response propositions [12,15,16]. More recent interoceptive models extend this account by proposing that mental imagery arises through reciprocal interactions between cortical imagery processes and afferent bodily signals, including autonomic and insular pathways [21,22]. Together, these perspectives suggest that guided imagery may produce both content-specific neuroendocrine responses and modality-independent physiological effects arising from shared interoceptive engagement.

Guided primarily by Lang's bio-informational theory and contemporary neuroscience models of stress and motivation, the present study used a randomized, counterbalanced crossover design to determine whether guided imagery content differentially influences subjective stress and neuroendocrine responses, thereby providing preliminary evidence to support more targeted selection of imagery interventions. It was hypothesized that calming imagery would reduce perceived stress and cortisol levels, reflecting enhanced parasympathetic activation and stress-related regulation, whereas achievement imagery would preferentially increase serotonin levels through greater engagement of motivational and reward-related pathways. A further hypothesis was that calming imagery would elicit greater increases in β-endorphin levels, consistent with its emphasis on relaxation and comfort, such that calming and achievement imagery would generate distinct psychophysiological profiles across subjective and neuroendocrine outcomes.

## 2 Materials and methods

### 2.1 Participants

Thirty-two male young adults, aged 18 to 25 years (20.8 ± 1.6 years) participated in this counterbalanced crossover study. Participant recruitment was conducted through university announcements, social media advertisements, and community health center flyers. Inclusion and exclusion criteria were defined for this study. The inclusion criteria were: (a) male aged 18-25 years, (b) self-reported elevated stress (Perceived Stress Scale [PSS-10] score ≥ 14), (c) access to a smartphone or computer with audio playback capability, (d) willingness to attend three laboratory visits for blood sample collection, and (e) ability to provide informed consent. In contrast, the exclusion criteria were: (a) current use of psychotropic medications within the past 4 weeks; (b) acute infectious illness (e.g., fever or influenza-like symptoms) within the week preceding blood sampling or current use of anti-inflammatory medications (e.g., non-steroidal anti-inflammatory drugs), as these conditions may influence circulating neuroendocrine biomarkers; (c) current engagement in regular meditation, yoga, or other formal relaxation practices (≥2 sessions per week); (d) diagnosis of a major psychiatric or chronic medical disorder; (e) current use of corticosteroid medications; (f) history of fainting or severe anxiety related to blood draws; and (g) participation in another stress-reduction intervention study within the past 3 months.

Medical history, psychiatric history, and current medication use were obtained by self-report during participant screening, using a standardized health questionnaire administered before enrollment. No structured clinical interview or medical record verification was performed.

All participants provided written informed consent prior to enrollment. The study was approved by the Institutional Review Board of the University of Jordan (Decision No. 529/2025, Ref No.19/2025/739), and was conducted in accordance with the Declaration of Helsinki.

### 2.2 Study design

This study employed a counterbalanced crossover design with two imagery modalities (calming, achievement). Each participant in the two intervention groups received calming and achievement images on different days, with the order of presentation counterbalanced between participants to control for sequence effects and inter-individual variability [23,24], to ensure statistical power [23], and to examine potential order effects. A control group (CG; n = 7) received no imagery intervention during the study period.

The intervention period spanned 5 days, with assessments conducted at baseline (Day 1), mid-intervention (Day 3), and post-intervention (Day 5). Day 3 served as a rest day between the two imagery modalities, with no imagery practice scheduled. Participants were randomly assigned to two groups according of sequence conditions imaging modality (M). First group Calming-first sequence (CAG; n = 13): Calming imagery on Day 2, Achievement imagery on Day 4. However, the second group Achievement-first sequence (ACG; n = 12): Achievement imagery on Day 2, Calming imagery on Day 4. Due to the nature of the intervention, only the researchers delivering the intervention recordings were aware of group allocation after assignment. Blood samples were collected on Days 3 and 5.

To characterize the temporal dynamics and modality-specific effects of imagery-based interventions, blood biomarkers representing three neuroendocrine systems were evaluated: cortisol for the hypothalamic-pituitary-adrenal (HPA) axis, serotonin for the serotonergic system, and β-endorphin for the endogenous opioid system. Blood samples were collected at baseline (day 1), after the first imagery modality (day 3), and after the second imagery modality (day 5). The control group was evaluated only on days 1 and 5.

The protocol, including study hypotheses, design, and analysis plan, was specified in advance as part of a formal grant/research proposal. However, this pilot study was not formally pre-registered on a public registry prior to data collection, owing to practical constraints at the time of study initiation. This is acknowledged as a limitation; the hypotheses tested were specified a priori rather than developed post hoc.

### 2.3 Guided imagery interventions

#### 2.3.1 Development of imagery scripts

Two guided imagery scenarios were developed based on Lang's bio-informational theory [15,16] and in collaboration with three independent experts (a clinical psychologist, a mental imagery researcher, and a mindfulness instructor) to ensure clarity. Each 10-min scenario incorporated multisensory details (visual, auditory, kinesthetic, and olfactory) for enhanced immersion and vividness. The scripts were recorded with high-quality audio, featuring a calm and soothing voice for the relaxation scenario and a more dynamic and motivating voice for the success scenario. Soft background music (ambient nature sounds for relaxation; uplifting instrumental music for success) was added at a low volume to further enhance immersion.

##### 2.3.1.1 Calming imagery script

The calming imagery script guided participants to imagine a peaceful natural environment (a quiet beach at sunset). The script emphasized relaxation, tranquillity, and sensory immersion, with specific attention to response propositions related to parasympathetic activation (slowed breathing, muscle relaxation, feelings of safety and calm). Example excerpt: "*Imagine yourself standing on a quiet, secluded beach at sunset. Feel the warm sand beneath your feet, soft and comforting. Notice the gentle rhythm of the waves as they roll onto the shore, creating a soothing, repetitive sound. With each breath, feel yourself becoming more and more relaxed. The warm breeze touches your skin, carrying the fresh scent of the ocean. You are completely safe, completely at peace …* ".

##### 2.3.1.2 Achievement imagery script

The achievement imagery script guided participants to imagine successfully completing an important personal goal (e.g., academic achievement, athletic success, career milestone). The script emphasized mastery, confidence, and positive performance outcomes, with specific attention to response propositions related to motivation, self-efficacy, and goal attainment. Example excerpt: "*Imagine yourself achieving an important goal you've been working toward. See yourself performing at your best focused, confident, and capable. Feel the sense of pride and accomplishment as you succeed. Notice how your body feels strong and energized. You are in control, moving forward with determination. This success is yours, earned through your effort and skill …* ".

#### 2.3.2 Intervention delivery

Immediately after their Day 1 visit, participants received the appropriate imagery audio file (calming or motivating, depending on their assigned sequence) via a secure, password-protected online platform (Google Drive). Participants were instructed to complete the first session on Day 2 and the second session on Day 4, at approximately the same time each day (morning or evening, according to their preference), in a quiet and comfortable location with minimal distractions, using headphones or earbuds for optimal audio quality.

Adherence to the imagery intervention was monitored through a brief electronic diary completed immediately after each imagery session. The diary assessed: (a) date and time of session, (b) duration, (c) location, (d) vividness of imagery (1-5 scale), (e) difficulty concentrating (1-5 scale), and (f) feelings of relaxation (1-5 scale).

### 2.4 Outcome measures

#### 2.4.1 Perceived Stress Scale (PSS-10) assessment

The Perceived Stress Scale [25] is a widely used 10-item self-report measure assessing the degree to which situations in one's life are appraised as stressful. Items are rated on a 5-point scale (0 = *never* to 4 = *very often*), with total scores ranging from 0 to 40. Higher scores indicate greater perceived stress. The PSS-10 was administered at baseline (Day 1) and post-intervention (Day 5).

#### 2.4.2 Analysis of blood biomarkers

Blood samples were collected by venipuncture into collection tubes (3 x 5 mL, for each participant) at three time points in the CAG and ACG on Day 1, 3, and 5, and on Days 1 and 5 in CG. All samples were collected in the morning (9:00–11:00 a.m.). Participants fasted for at least 2 h before sampling and avoided caffeine, alcohol, and strenuous exercise for 24 h prior to each visit.

To perform the blood cortisol (BC) analyses, one of the three blood samples was collected in a serum separator tube (SST; Becton Dickinson). The blood samples (BC) were coagulated for 30 min at room temperature and then centrifuged at 1500 g for 15 min at 4°C. The serum was aliquoted into cryotubes and stored at −80°C. Cortisol (ng/mL) concentrations were quantified by enzyme-linked immunosorbent assay (ELISA kit for cortisol, DRG International, Springfield, NJ). The assay has a sensitivity of 2.5 ng/mL and intra- and inter-assay coefficients of variation (CV) of less than 5% and 8%, respectively.

The second tube was used for blood β-endorphin (BE) analysis. Blood samples (5 mL) were collected in refrigerated EDTA tubes containing aprotinin (500 KiU/mL of blood). The tubes were immediately placed on ice and centrifuged at 4°C (2000 × g for 15 min). The plasma was aliquoted into cryotubes and stored at −80°C until analysis. BE (pg/mL) concentrations were measured by enzyme immunoassay (EIA β-endorphin kit, Peninsula Laboratories International, San Carlos, CA). This assay has a sensitivity of 15 pg/mL and intra- and inter-assay CVs of less than 5% and 10%, respectively.

The third tube was for the analysis of blood serotonin (BS). The samples were collected in EDTA tubes, homogenized by gentle inversion, and stored at −80°C. BS (ng/mL) concentrations were quantified by high-performance liquid chromatography with electrochemical detection (HPLC-ECD; Waters Corporation, Milford, MA). The assay exhibited a sensitivity of 5 ng/mL and intra-assay and inter-assay CV of less than 4% and 7%, respectively.

### 2.5 Statistical analysis

All statistical analyses were performed in R software (version 4.6.1; R Foundation for Statistical Computing, Vienna, Austria) within RStudio, using the packages lme4 and lmerTest for mixed-effects modeling, emmeans for estimated marginal means and post-hoc contrasts, effectsize for partial eta-squared, performance and DHARMa for model diagnostics, and car, FSA, and rstatix for baseline comparisons. Descriptive statistics are reported as mean ± SD unless otherwise specified. The significance threshold was set at p < .05 (two-tailed) throughout.

Baseline equivalence: Equivalence at Day 1 across the three groups (CG, untreated control; CAG, calming-then-achievement; ACG, achievement-then-calming) was assessed for continuous demographic variables (age, academic year) with one-way ANOVA, complemented by the Kruskal-Wallis test as the primary result whenever the normality of ANOVA residuals was violated (Shapiro-Wilk test); significant omnibus tests were followed by Tukey HSD or Dunn post-hoc comparisons (Holm-adjusted). Categorical demographic variables (prior imagery experience, chronic illness, medication affecting the HPA axis, psychiatric diagnosis history) were compared with Fisher's exact test across the three groups, with pairwise Fisher post-hoc comparisons (Holm-adjusted) where the omnibus test was significant. Baseline equivalence for perceived stress and the three biomarkers was assessed from the Day-1 estimated marginal means of the confirmatory linear mixed models described below, so that the baseline comparison uses the same model as the hypothesis tests.

Confirmatory analyses: Because the study combines a counterbalanced within-subject crossover (CAG, ACG) with an independent, non-randomized control group (CG) assessed at Day 1 and Day 5 only, all repeated outcomes (perceived stress [PSS-10 total score], cortisol, β-endorphin, serotonin) were analyzed with linear mixed models (LMM), to accommodate the unbalanced assessment schedule and to retain all available data (rather than a subset of timepoints or participants) in a single model. All models included a random intercept for participant, were fitted by restricted maximum likelihood (REML), and used Satterthwaite's approximation for denominator degrees of freedom. Two complementary sets of models were fitted for each outcome.

- An omnibus model with Test (Day 1, 3, 5) × Group (CG, CAG, ACG) as fixed effects, testing whether trajectories differed between the two active sequences and the untreated control condition, while accounting for the fact that CG and perceived stress were assessed only on days 1 and 5.
- A crossover-specific model, restricted to the two active groups (CAG, ACG) and to the two post-baseline assessments (Day 3, Day 5), with Modality (calming vs. achievement imagery, irrespective of order), Period (first vs. second exposure within the crossover), and Sequence (CAG vs. ACG, i.e., the order in which the two modalities were administered) as fixed effects. This model isolates the within-person, modality-specific contrast proper to the crossover design, independent of administration order.

For each model, Type III F-tests (Satterthwaite) were used to test fixed effects, and partial eta-squared (η^2^p) was computed from each F-test as the associated effect size. Marginal and conditional R^2^ (variance explained by fixed effects alone, and by fixed and random effects combined, respectively) were computed for the omnibus models. Post-hoc pairwise comparisons used estimated marginal means with Bonferroni correction (Holm correction was also computed and yielded equivalent conclusions).

Model diagnostics: For each model, residual normality was assessed with the Shapiro-Wilk test and visual inspection of QQ-plots, homoscedasticity was tested formally, and simulation-based residual diagnostics (DHARMa: uniformity/Kolmogorov-Smirnov test, dispersion test, outlier test) were used as an additional check of model adequacy; influential observations were screened with Cook's distance. Departures from normality or homoscedasticity are reported alongside the corresponding results and considered when interpreting the findings.

Multiple comparisons: Perceived stress, cortisol, β-endorphin, and serotonin were tested without a single pre-specified primary outcome; no correction was applied across these four outcomes.

## 3 Results

### 3.1 Participant characteristics and baseline equivalence

Thirty-two participants completed the study without any dropouts and were randomly assigned to the experimental groups (CAG, n = 13; ACG, n = 12), and 7 constituted a concurrently assessed, untreated control group (CG). The sample consisted exclusively of men, with a mean age of 20.88 ± 1.62 years. Baseline perceived stress scores indicated moderate to high stress levels (PSS-10: 28.81 ± 3.57), confirming successful recruitment of a stressed population appropriate for stress reduction interventions (Table 1).

**Table 1:** Participant characteristics and baseline equivalence (Day 1).

Characteristic	Total (N = 32)	CAG (n = 13)	ACG (n = 12)	**CG (n = 7)**	Test (3 groups)
Age (years)	20.88 ± 1.62(18–25)	21.08 ± 1.85	20.67 ± 1.44	20.86 ± 1.68	Kruskal-Wallis χ^2^(2) = .13, *p* = .939
Academic year (1–4)	3.15 ± .83 (1–4)	3.23 ± .60	3.25 ± .87	2.71 ± 1.11	Kruskal-Wallis χ^2^(2) = 1.47, *p* = .479
Prior imagery experience (Yes)	16 (50.0%)	7 (53.8%)	7 (58.3%)	2 (28.6%)	Fisher's exact *p* = .539
Chronic illness diagnosis (Yes)	0 (.0%)	0 (.0%)	0 (.0%)	0 (.0%)	Not applicable (no cases)
Medication affecting HPA axis (Yes)	1 (3.1%)	1 (7.7%)	0 (.0%)	0 (.0%)	Fisher's exact *p* = 1.000
Psychiatric diagnosis history (Yes)	1 (3.1%)	1 (7.7%)	0 (.0%)	0 (.0%)	Fisher's exact *p* = 1.000
PSS-10 total (PSSpre 1–10 total score)	28.81 ± 3.57	29.62 ± 4.13	27.42 ± 3.37	29.71 ± 2.14	CG–CAG *p* = 1.00; CG–ACG *p* = .565; CAG–ACG *p* = .815
Cortisol (ng/mL)	119.68 ± 29.67	117.02 ± 36.23	122.68 ± 27.34	119.46 ± 22.81	CG–CAG *p* = 1.00; CG–ACG *p* = 1.00; CAG–ACG *p* = 1.00
β-Endorphin (pg/mL)	437.20 ± 156.50	368.14 ± 149.89	413.64 ± 110.90	605.86 ± 120.11	a CG–CAG *p* = .002; a CG–ACG *p* = .019; CAG–ACG *p* = 1.00
Serotonin (ng/mL)	96.18 ± 67.35	70.37 ± 34.28	112.34 ± 74.08	116.41 ± 92.64	CG–CAG *p* = .806; CG–ACG *p* = 1.00; CAG–ACG *p* = .713

Mean ± Sd. Continuous demographic variables: one-way ANOVA (Age: F(2,29) = .19, p = .828, η^2^ = .013; Academic year: F(2,29) = 1.10, p = .347, η^2^ = .070) with Kruskal-Wallis reported as the primary test given non-normal ANOVA residuals for academic year (Shapiro-Wilk on residuals, p = .013; Age residuals were normal, p = .542); Tukey post-hoc comparisons were non-significant for both variables (all p ≥ .38). Categorical demographic variables: Fisher's exact test (3 groups). Baseline (Day 1) pairwise comparisons for perceived stress and the three biomarkers are the estimated-marginal-mean contrasts (Bonferroni-adjusted) from the omnibus Test × Group linear mixed models.

a p < .05.

For the four repeated biomarkers, a formal three-group baseline comparison is available from the Day-1 estimated marginal means of the omnibus models (Bonferroni-adjusted). This comparison shows that the three groups were not fully equivalent at baseline (Table 1): β-endorphin was significantly higher in CG than in either active group (CG vs. ACG: β = 192.2, 95% CI [56.1, 328.3], p = .019; CG vs. CAGACG: β = 237.7, 95% CI [103.6, 371.8], p = .002), whereas no significant baseline group differences were found for perceived stress, cortisol, or serotonin (all p ≥ .57). This baseline imbalance in β-endorphin should be acknowledged as a limitation when interpreting the corresponding group-by-time results. However, these results confirm successful randomization between ACG and CAG and support the validity of subsequent comparisons.

### 3.2 Modality-specific effects on neuroendocrine biomarkers

To isolate the within-person, modality-specific effect proper to the crossover design, a dedicated LMM was fitted for each biomarker in the two active groups only (CG excluded, as it did not receive either imagery modality), restricted to the two post-baseline assessments (Day 3, Day 5), with Modality (calming vs. achievement imagery, irrespective of order), Period (first vs. second exposure), and Sequence (CAG vs. ACG) as fixed effects.

Regarding cortisol, no significant effect of modality, period, or sequence (Table 2). Estimated mean cortisol was 127 ± 6.1 ng/mL following calming imagery and 123 ± 6.1 ng/mL following achievement imagery (β = 4.07, p = .610).

**Table 2:** Modality-specific effects (Day 3–Day 5, CAG/ACG only; Modality + Period + Sequence + (1|ID)).

Biomarker	Effect	β (Δ)	SE	95% CI	F	df	p	η^2^p
Cortisol (ng/mL)	Modality (Calming–Achievement)	4.07	7.87	[–12.2, 20.3]	.27	1, 23	.610	.011
	Period	—	—	—	.89	1, 23	.354	.037
	Sequence	—	—	—	.98	1, 23	.333	.041
β-Endorphin (pg/mL)	Modality (Calming–Achievement)	12.1	41.0	[–72.7, 96.9]	.09	1, 46	.769	.002
	Period	—	—	—	.19	1, 46	.669	.004
	Sequence	—	—	—	.64	1, 46	.427	.014
Serotonin (ng/mL)	Modality (Calming–Achievement)	−66.5	27.4	[–123.2, −9.8]	5.88	1, 46	.019^a^	.113^a^
	Period	—	—	—	1.43	1, 46	.238	.030
	Sequence	—	—	—	6.60	1, 46	.014^a^	.125^a^

Linear mixed models: biomarker ∼ Modality + Period + Sequence + (1|ID), random intercept for participant, REML, Satterthwaite degrees of freedom. Modality = calming vs. achievement imagery, irrespective of the order in which it was administered. Period = first vs. second exposure within the crossover (i.e., Day 3 vs. Day 5, regardless of which modality was given). Sequence = CAG vs. ACG, i.e., the order in which the two modalities were administered (calming-then-achievement vs. achievement-then-calming). β = fixed-effect estimate for the Modality contrast (Calming – Achievement); a negative value indicates higher levels following achievement imagery. SE = standard error of β; 95% CI = 95% confidence interval of β, computed as β ± t(df,.975) × SE. F, df, and p refer to the Type III test of each fixed effect. η^2^p = partial eta-squared, computed from F and its degrees of freedom [η^2^p = (F×df1)/(F×df1+df2)]. N = 25 participants (CAG, n = 13; ACG, n = 12), 50 observations. CG did not receive either imagery modality and is not included here.

a p < .05.

Similarly, for β-endorphin, there was no significant effect of modality, period, or sequence (Table 2). Estimated mean β-endorphin was comparable following calming (607 ± 29 pg/mL) and achievement imagery (595 ± 29 pg/mL; β = 12.1, p = .770).

In contrast, regarding serotonin, a significant effect of modality was found (Table 2): serotonin was higher following achievement imagery (189 ± 19 ng/mL) than calming imagery (123 ± 19 ng/mL; β = −66.5 [Calming – Achievement], 95% CI [–123.2, −9.8], p = .019, η^2^p = .113). A significant sequence effect was also found (η^2^p = .125, p = .014). Period was not significant (Fig. 1).

### 3.3 Time and sequence effects on neuroendocrine biomarkers

To describe each biomarker's full trajectory and test for group effects including the untreated control condition, an omnibus LMM was fitted per biomarker with Test (Day 1, 3, 5) × Group (CAG, ACG, CG) as fixed effects. The control group contributed data at Day 1 and Day 5 only.

Regarding cortisol, no significant effect of time, group, or their interaction (Table 3). No within- or between-group contrast reached significance at any timepoint (all p ≥ .26; Fig. 2A).

**Table 3:** Omnibus time × group effects (Test × Group + (1|ID), N = 32).

Biomarker	Effect	F	df	p	η^2^p	R^2^c	R^2^m
Cortisol (ng/mL)	Time	1.11	2, 53.8	.336	.04	.138	.047
	Group	.85	2, 35.0	.437	.05		
	Time × Group	.38	3, 53.8	.767	.02		
β-Endorphin (pg/mL)	Time	12.64	2, 51.2	< .001^b^	.33	.357	.322
	Group	4.13	2, 32.5	.025^a^	.20		
	Time × Group	1.78	3, 51.2	.162	.09		
Serotonin (ng/mL)	Time	22.57	2, 50.1	< .001^b^	.47	.523	.509
	Group	16.20	2, 31.5	< .001^b^	.51		
	Time × Group	7.95	3, 50.1	< .001^b^	.32		

Omnibus effects of Test (assessment day: Day 1, Day 3, Day 5) and Group (CG, untreated control, n = 7, assessed at Day 1 and Day 5 only; CAG, calming-then-achievement, n = 13; ACG, achievement-then-calming, n = 12) on each biomarker, from linear mixed models (biomarker ∼ Test × Group + (1|ID), random intercept for participant, REML, Satterthwaite degrees of freedom). N = 32 participants, 89 observations (7 fewer than the theoretical maximum of 96 owing to the absence of a Day 3 assessment in CG). F, df, and p refer to the Type III test of each fixed effect. η^2^p = partial eta-squared. R^2^c = conditional R^2^ (variance explained by fixed and random effects); R^2^m = marginal R^2^ (variance explained by fixed effects only); both computed with performance:r2.

a p < .05.

b p < .001.

However, β-Endorphin levels showed significant main effects of time (η^2^p = .33, p < .001) and group (η^2^p = .20, p = .025), but no significant time × group interaction (η^2^p = .09, p = .162). Both active groups showed a significant rise from Day 1 to Day 3 that plateaued thereafter (Fig. 1), whereas CG showed no significant change over the study period (β = −25.7 pg/mL, 95% CI [–175.8, 124.5], p = .733). As noted, CG had significantly higher β-endorphin than both active groups at Day 1 (Fig. 1); by Day 5 no between-group differences remained (all p = 1.0; Fig. 2B).

**Fig. 1:**
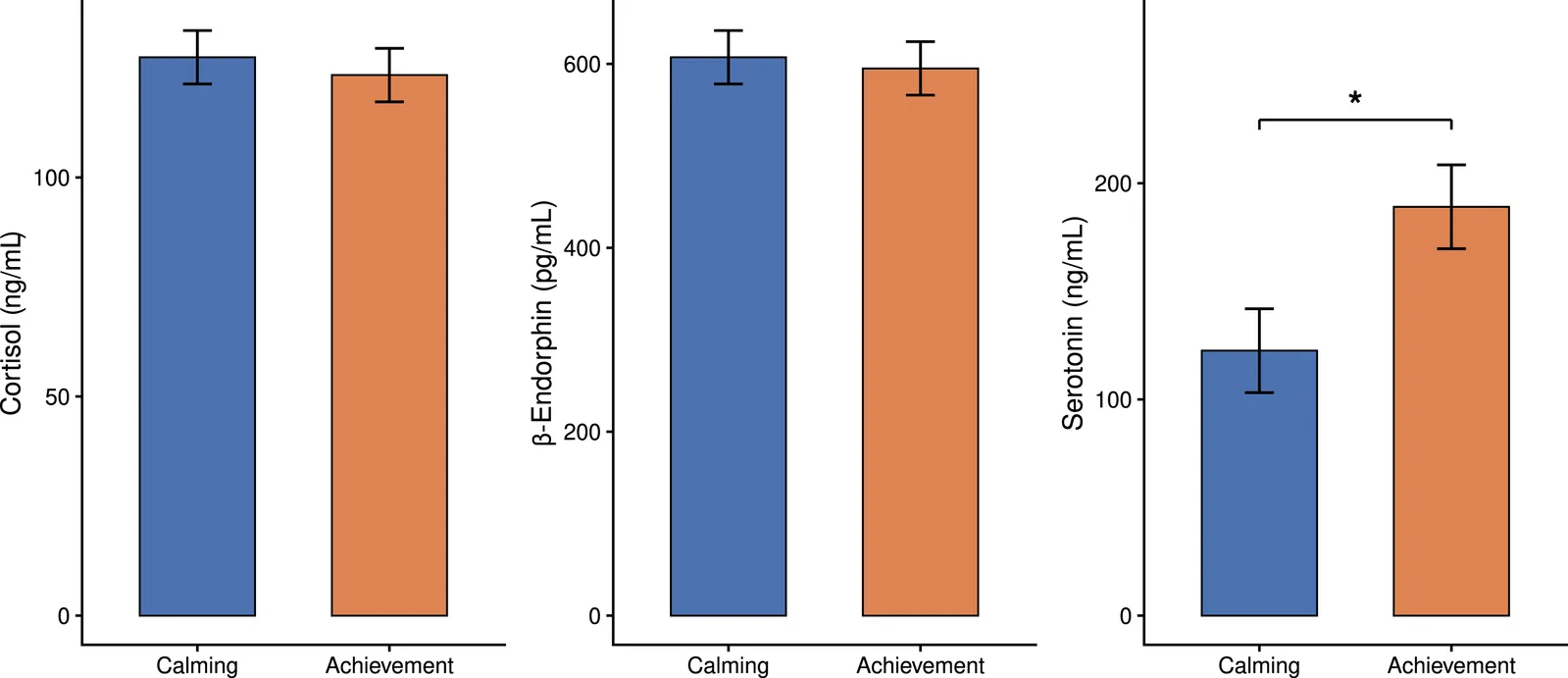
Modality-specific effect on cortisol, β-endorphin, and serotonin, pooling data across sequence and period (Day 3 and Day 5 combined, CAG/ACG groups only). *Note:* Bars represent estimated marginal means ± SE from the crossover-specific linear mixed models (biomarker ∼ Modality + Period + Sequence + (1|ID)). Calming and achievement conditions each combine one observation per participant (from whichever day that participant received that modality), so that each bar reflects both sequences equally. * = p < .05 (Bonferroni/Holm-adjusted contrast).

**Fig. 2:**
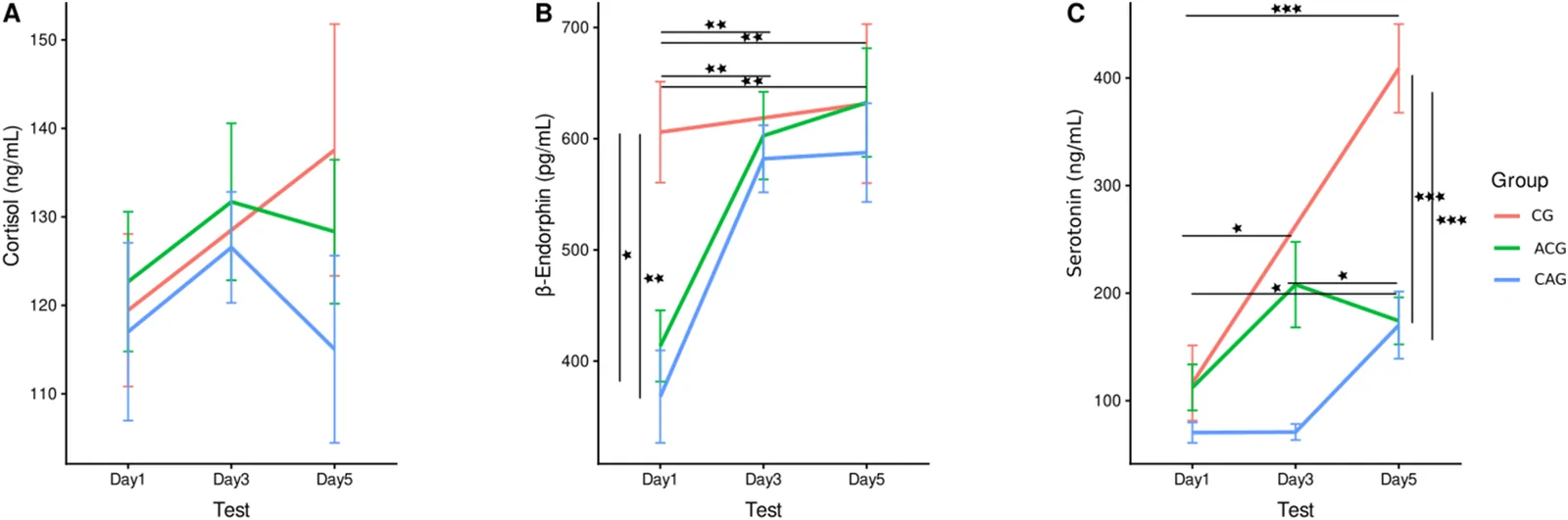
Trajectoir of blood cortisol, β-endorphin and serotonin across time (Day 1, Day 3, Day 5) following the first exposure to the intervention of both imaging modalities. *Note:* CAG: group calming-achievement; ACG: group achievement-calming; CG: control group. Cortisol (A), β-endorphin (B), and serotonin (C) across the three study groups (CG, CAG, ACG). Points and error bars represent estimated marginal means ± SE from the omnibus Test × Group linear mixed models reported in Table 3. CG was assessed at Day 1 and Day 5 only (no Day 3 imagery session). Asterisks placed above a line segment, in the color of the corresponding group, denote a statistically significant within-group change between the two connected timepoints (Bonferroni-adjusted pairwise contrast). Asterisks above a horizontal bracket denote a statistically significant between-group difference at the indicated day (Bonferroni-adjusted pairwise contrast). Significance codes: *p < .05, **p < .01, ***p < .001.

For serotonin, there were significant main effects of time (η^2^p = .47, p < .001) and group (η^2^p = .51, p < .001), as well as significant time × group interaction (η^2^p = .32, p < .001; Table 3). CG showed a large, significant increase from Day 1 to Day 5 (β = −292.5 ng/mL, 95% CI [–385.8, −199.2], p < .0001), exceeding the increase observed in either active group and reaching a significantly higher Day 5 value than both ACG and CAG (Fig. 1). At Day 3, ACG (achievement first) had significantly higher serotonin than CAG (calming first) (β = 137.0, 95% CI [66.7, 207.3], p < .001), consistent with §3.2; this difference was no longer present at Day 5 (Fig. 2C).

### 3.4 Changes in perceived stress

An omnibus LMM (Test [Day 1, Day 5] × Group [CAG, ACG, CG] + (1|ID)) on the PSS-10 total score showed no significant main effect of time, group, or their interaction (Table 4). No within-group change from Day 1 to Day 5 was significant in any group (β ranged from −.43 to .46, all *p* ≥ .65), and no between-group contrast reached significance at either timepoint (all *p* ≥ .43, Bonferroni-adjusted).

**Table 4:** Omnibus time × group effects on perceived stress (Test × Group + (1|ID), N = 32).

Effect	F	df	p	η^2^p	R^2^c	R^2^m
Time	.005	1, 29	.947	< .001	.642	.070
Group	1.43	2, 29	.256	.09		
Time × Group	.17	2, 29	.846	.01		

Effects of Test (Day 1 vs. Day 5; the PSS-10 was not administered on Day 3) and Group (CG, n = 7; CAG, n = 13; ACG, n = 12) on the PSS-10 total score, from a linear mixed model (PSS-10 ∼ Test × Group + (1|ID), random intercept for participant, REML, Satterthwaite degrees of freedom). N = 32 participants, 64 observations. F, df, and p refer to the Type III test of each fixed effect. η^2^p = partial eta-squared. R^2^c = conditional R^2^ (fixed + random effects); R^2^m = marginal R^2^ (fixed effects only). No effect or contrast reached statistical significance.

Residuals of this model were approximately normal (Shapiro-Wilk W = .974, p = .196) and homoscedastic (p = .359); four influential observations were flagged by Cook's distance and retained in the reported model. Overall, this mixed-model analysis of the complete repeated-measures structure does not support a differential effect of imagery sequence, or of either modality relative to no intervention, on perceived stress.

## 4 Discussion

The present study investigated whether the perceived stress and neuroendocrine effects of guided imagery depend on imagery content, by comparing calming and achievement imagery within a randomized, counterbalanced crossover design. This comparison was intended to distinguish physiological responses common to guided imagery from those associated specifically with relaxation-oriented versus motivationally activating content, thereby clarifying whether imagery effects relevant to stress regulation depend on the regulatory goal of the script. Demonstrating such content specificity would have practical implications for selecting imagery interventions according to therapeutic objectives, rather than assuming that all imagery scripts produce equivalent biological effects.

The principal finding was that serotonin concentrations were higher following achievement imagery than following calming imagery within the active crossover groups, whereas β-endorphin increased over time without a significant modality-specific effect, and cortisol remained unchanged. Importantly, these neuroendocrine responses were not accompanied by measurable changes in perceived stress during the five-day intervention. Together, these findings suggest that guided imagery did not produce a uniform pattern across the measured outcomes, and that some neuroendocrine responses may vary according to imagery content, consistent with Lang's bio-informational theory and contemporary interoceptive models of mental imagery [15,16,21,22]. Collectively, the findings reveal a partial dissociation between neuroendocrine systems, consistent with previous evidence that guided imagery can produce selective rather than uniform psychophysiological effects depending on the characteristics of the intervention [[12], [13], [14],^19^]. While these findings should be regarded as hypothesis-generating and require confirmation in adequately powered, pre-registered trials, they provide preliminary evidence that different guided imagery modalities may be associated with distinct patterns of peripheral neuroendocrine response.

The most notable finding of the present study was the higher serotonin concentration observed following achievement imagery. In the crossover-specific model, serotonin concentrations were significantly higher following achievement imagery than following calming imagery after adjustment for period and sequence (p = .019). This finding suggests a potential modality-specific serotonergic response to achievement-oriented imagery. However, a significant sequence effect was also observed (p = .014), indicating that the order in which the two imagery modalities were administered may have influenced serotonin concentrations. Therefore, although the modality effect remained significant after statistical adjustment, it should not be interpreted as entirely independent of administration order. Given the small pilot sample and the substantial serotonin increase observed in the untreated control group, these findings should be considered preliminary and require confirmation in larger, adequately powered crossover trials. They are nonetheless consistent with the possibility that achievement-oriented imagery engages physiological processes related to motivation and goal-directed behavior. Although serotonin has traditionally been associated with affective regulation, contemporary evidence indicates that it also plays a central role in motivation, reward anticipation, behavioral persistence, effort allocation, and adaptive goal-directed behavior [[26], [27], [28], [29]]. Achievement imagery requires participants to mentally simulate successful goal attainment, competence, and mastery, processes likely to engage these motivational networks more strongly than relaxation-focused imagery [15,16]. Consequently, the present findings extend previous behavioral evidence by suggesting that distinct guided imagery scripts may differentially influence measurable neuroendocrine responses, in addition to subjective psychological processes [[12], [13], [14]].

The selective serotonergic response observed here can be interpreted within both Lang's established bio-informational theory and contemporary interoceptive models of mental imagery [21,22]. Lang's theory proposes that mental imagery activates physiological response networks according to its semantic content, rather than merely eliciting a nonspecific relaxation response [15,16]. The present findings provide preliminary peripheral biomarker evidence that imagery content differentially engages physiological systems according to its motivational significance, extending previous behavioral findings by suggesting content-specific physiological activation during guided imagery, alongside its well-established psychological effects [[12], [13], [14]]. While Lang's theory accounts for the modality-specific serotonergic response, it does not fully explain the comparable β-endorphin increase observed across both imagery conditions, suggesting that additional physiological mechanisms may contribute to the effects of guided imagery.

An additional explanation is offered by contemporary interoceptive models of mental imagery [21,22]. Rather than viewing imagery solely as a top-down cognitive process, these models propose that imagery emerges through reciprocal interactions between cortical imagery networks and afferent autonomic signals, particularly within the posterior insular cortex. From this perspective, the comparable β-endorphin response observed following both calming and achievement imagery may reflect shared engagement of interoceptive and autonomic processes elicited during immersive imagery practice, irrespective of specific content. Under this framework, guided imagery may involve the integration of top-down cognitive representations with bottom-up interoceptive signaling, with different neuroendocrine systems varying in their sensitivity to imagery content. The higher serotonin concentration following achievement imagery may then also reflect the direction of autonomic mobilization - greater activation during achievement imagery relative to calming imagery - rather than semantic-content specificity alone; however, autonomic activity was not measured directly in the present study, and this interpretation remains speculative. Although these mechanistic interpretations remain preliminary given the pilot nature of the study, the interoceptive framework offers a biologically plausible, parsimonious explanation for the modality-independent β-endorphin response and warrants further investigation using multimodal neurophysiological techniques, including concurrent autonomic measures.

β-Endorphin increased significantly over time in both active imagery groups, but the absence of a significant time × group interaction and the baseline imbalance in this biomarker between the control and intervention groups prevent this change from being attributed specifically to guided imagery. Contrary to hypothesis, calming and achievement imagery produced comparable β-endorphin responses. As an endogenous opioid peptide, β-endorphin contributes to analgesia, emotional regulation, stress adaptation, and subjective well-being, and has been associated with relaxation, positive affect, and stress reduction [7,8,30,31]. The rise in β-endorphin observed over time may therefore reflect temporal, nonspecific, or general engagement-related influences rather than a modality-specific effect, a possibility further supported by evidence linking positive affect and reward-related processing to endogenous opioid activity [7,30,32]. Taken together, this evidence suggests that guided imagery may influence peripheral physiological processes associated with well-being and stress adaptation, although the clinical significance of the observed β-endorphin changes remains uncertain. Accordingly, the choice of imagery modality may be guided primarily by therapeutic objectives and participant preference, rather than by expectations of differential opioid activation [33,34].

In contrast to serotonin and β-endorphin, cortisol concentrations remained unchanged throughout the intervention, regardless of modality, timepoint, group, or sequence. This finding indicates that the five-day guided-imagery program did not produce a measurable change in circulating cortisol. Previous studies examining guided imagery and related mind–body interventions have reported variable cortisol responses, with changes potentially depending on intervention duration and repeated practice [17,18,35]. Cortisol secretion is also influenced by numerous physiological and methodological factors, including circadian variation, anticipatory responses, individual stress reactivity, and sampling protocols, all of which may reduce sensitivity to short-term behavioral interventions [[36], [37], [38]]. Because biomarkers were measured only once per assessment day, within a fixed 9–11 a.m. window, each measurement represents only a snapshot of endocrine activity and cannot characterize within-day secretion dynamics or diurnal patterns - a limitation that is especially relevant for cortisol, given its steep decline during this window following the post-awakening peak. The absence of a cortisol response should therefore not be interpreted as evidence that guided imagery lacks physiological effects, but rather suggests that cortisol may be less sensitive than serotonin or β-endorphin to short-term, modality-specific neuroendocrine adaptations under the present sampling protocol.

Perceived stress did not change significantly over the study period, despite changes in some neuroendocrine markers. This pattern may indicate that subjective and physiological responses were not closely coupled in this short pilot study, consistent with previous evidence that emotional and physiological stress responses do not always correspond [39]. This interpretation should, however, remain tentative: the biomarker responses were heterogeneous across systems, and serotonin concentrations also increased substantially in the untreated control group which limits confidence in any single explanatory account of the biomarker–stress dissociation. Self-reported stress reflects a broad cognitive appraisal shaped by accumulated daily experience and may require a longer intervention period, or more frequent sampling, before meaningful within-protocol changes become detectable. A further, methodological explanation is also plausible: the PSS-10 was administered with its standard, unmodified recall period at both Day 1 and Day 5, and this period is considerably longer than the 5-day interval between the two administrations. The instructed appraisal window therefore overlapped substantially between assessments, which may itself have limited the instrument's sensitivity to detect short-term, within-protocol change; a state-level or momentary stress measure with a recall period matched to the assessment interval may be more appropriate in future short-duration intervention studies.

The present findings have potential implications for the development of personalized guided imagery interventions. Although both imagery modalities appear capable of engaging physiological pathways associated with well-being, achievement imagery may be particularly appropriate when interventions aim to enhance motivation, behavioral persistence, and goal-directed performance, whereas calming imagery may remain preferable when the primary therapeutic objective is relaxation or emotional regulation. These observations are compatible with a precision-intervention framework, in which imagery content is selected according to the intended clinical or performance target rather than assumed to be interchangeable [34]. Given the pilot nature of the study, its modest sample size, and the absence of direct behavioral performance outcomes, these practical implications should be considered preliminary until confirmed in larger, pre-registered randomized trials.

### 4.1 Limitations and future prospects

The present study has several strengths. The randomized, counterbalanced crossover design, in which each participant served as their own control, reduced inter-individual variability and increased sensitivity to detect modality-specific neuroendocrine responses. The inclusion of objective biological markers alongside perceived stress provided a multidimensional assessment of guided imagery effects, and the use of complementary analytical approaches - a crossover-specific model isolating the within-person modality contrast, alongside omnibus linear mixed models incorporating the full repeated-measures dataset and an untreated comparison group - enhanced the robustness and transparency of the findings relative to original analysis.

Nevertheless, several limitations should be acknowledged. As a pilot study, the modest sample size limits statistical power and the precision of effect estimates, and no a priori power analysis was conducted to justify the original target enrollment. The untreated control group was also small (n = 7) and non-randomized; β-endorphin concentrations differed significantly between the control and intervention groups at baseline, limiting causal interpretation of the omnibus group comparisons, and serotonin increased more in this group than in either active group by Day 5, a pattern that cannot presently be attributed to the intervention, or ruled out as reflecting an uncontrolled temporal or diurnal process. In addition, the significant sequence effect on serotonin indicates that administration order may have influenced the results despite the counterbalanced design, raising the possibility of a carry-over effect that a longer washout period between imagery conditions could help address in future work. The findings are further limited to healthy young men, restricting generalizability to women, older adults, clinical populations, and individuals with different baseline stress profiles. The intervention comprised only two guided imagery sessions over five days; consequently, the minimum exposure required to produce reliable biological or psychological effects remains unknown, and this dosing decision was not itself empirically justified or tested.

Furthermore, no direct neurophysiological measures (e.g., autonomic indices or neuroimaging) were obtained to test the proposed neurobiological mechanisms underlying the observed biomarker responses, and acute infection or anti-inflammatory medication use was not formally screened at each assessment visit, leaving open a further uncontrolled source of biomarker variability. The interpretation of circulating serotonin and β-endorphin should also be made cautiously, because peripheral concentrations do not necessarily reflect central neurotransmitter or opioid activity and may be influenced by multiple systemic physiological processes. In addition, behavioral outcomes were not assessed; therefore, the observed neuroendocrine responses cannot be linked directly to changes in motivation, performance, or psychological functioning.

Multiple outcomes (perceived stress, cortisol, β-endorphin, serotonin) were examined without formal adjustment for multiple comparisons and without a single pre-specified primary outcome, increasing the risk of Type I error; accordingly, the observed findings - including the serotonin modality effect - should be regarded as hypothesis-generating and confirmed in adequately powered studies with pre-specified primary outcomes. The mechanistic interpretations offered above remain inferential and should be tested directly in larger, adequately powered, pre-registered studies incorporating multimodal neuroimaging or peripheral autonomic recording, longer intervention periods, and more diverse populations.

Despite these limitations, the present study provides novel preliminary evidence that imagery content may differentially influence neuroendocrine systems, supporting the feasibility of future mechanistic trials examining content-specific effects of guided imagery. Future research should examine longer intervention periods, larger and more diverse populations, a fully randomized control condition, and multimodal assessment strategies combining repeated or fixed-clock-time biomarker sampling with autonomic measures (e.g., heart rate variability) and functional neuroimaging, to better characterize the biological mechanisms underlying different guided imagery modalities. Such studies will help determine whether imagery content can selectively modulate distinct neuroendocrine and interoceptive-autonomic mechanisms, and thereby support the development of more precise, mechanism-based psychological interventions.

## 5 Conclusions

This pilot study suggests that peripheral neuroendocrine responses to guided imagery may vary according to imagery content. Within the active crossover groups, serotonin concentrations were higher following achievement imagery than following calming imagery after adjustment for period and sequence, although a significant sequence effect indicates that administration order may also have influenced serotonin concentrations. β-Endorphin increased significantly over time in both active groups, without a significant modality-specific or time × group effect, whereas cortisol concentrations and perceived stress remained unchanged throughout the intervention. Given the small sample size, the absence of a pre-specified primary outcome among multiple biomarkers tested, the baseline β-endorphin imbalance, the small and non-randomized control group, and the substantial serotonin increase observed in this untreated control group, these findings should be regarded as hypothesis-generating and confirmed in larger, adequately powered, pre-registered randomized trials.

## CRediT authorship contribution statement

**Nurwina Anuar:** Writing – review & editing, Writing – original draft, Validation, Supervision, Project administration, Methodology, Investigation, Funding acquisition, Formal analysis, Data curation, Conceptualization. **Manal M. Bayyat:** Writing – review & editing, Writing – original draft, Validation, Supervision, Project administration, Methodology, Investigation, Funding acquisition, Formal analysis, Data curation, Conceptualization. **Ibrahim M. Dabayebeh:** Writing – review & editing, Validation, Supervision, Methodology, Conceptualization. **Shazli Ezzat Ghazali:** Writing – review & editing, Writing – original draft, Formal analysis. **Zainab H. Abumuaili:** Writing – review & editing, Validation, Methodology, Formal analysis, Data curation. **Tala Al-Hadid:** Writing – review & editing, Validation, Methodology. **Haidar Djemai:** Writing – review & editing, Writing – original draft, Validation, Methodology, Conceptualization, Formal analysis, Visualization.

## Funding

This work was supported by the 10.13039/501100004515Universiti Kebangsaan Malaysia, Malaysia [grant number DPK-GPS-JORDAN-2024-026]; and the 10.13039/501100005712University of Jordan (JU), Jordan [grant number 107-/2024-2025].

## Declaration of competing interest

The authors declare the following financial interests/personal relationships which may be considered as potential competing interests: Nurwina Anuar & Manal M. Bayyat reports financial support was provided by Universiti Kebangsaan Malaysia & The University of Jordan. If there are other authors, they declare that they have no known competing financial interests or personal relationships that could have appeared to influence the work reported in this paper.
